# Freedom and Confinement: Patients' Experiences of Life with Home Haemodialysis

**DOI:** 10.1155/2014/252643

**Published:** 2014-12-18

**Authors:** C. Vestman, M. Hasselroth, M. Berglund

**Affiliations:** ^1^Department of Dialysis, Skaraborgs Hospital, 541 85 Skövde, Sweden; ^2^School of Health and Learning, University of Skövde, Box 408, 541 28 Skövde, Sweden

## Abstract

Patients with chronic end stage renal disease need dialysis to survive; however, they also need a treatment that suits their life situation. It is important that healthcare providers provide reliable, up-to-date information about different dialysis treatment options. Since home haemodialysis is a relatively new treatment, it is necessary to gather more knowledge about what the treatment entails from the patient's perspective. The aim of this study was to describe patients' experiences of having home haemodialysis. To gain access to the patients' experiences, they were asked to write narratives, which describe both their good and bad experiences of life with the treatment. The narratives were analysed with a qualitative method. The results of this analysis are subdivided into five themes: freedom to be at home and control their own treatment, feeling of being alone with the responsibility, changes in the home environment, need for support, and security and well-being with home haemodialysis. The conclusion is that home haemodialysis provides a certain level of freedom, but the freedom is limited as the treatment itself is restrictive. In order to improve patients' experiences with home haemodialysis, more research based on patients' experiences is needed and it is necessary to involve the patients in the development of the care.

## 1. Introduction

To suffer from chronic end stage renal disease means that the person becomes dependent on a life-long treatment in order to survive [1]. Today there are many different treatment regimens such as haemodialysis (HD), home haemodialysis (HHD), peritoneal dialysis (PD), and kidney transplant (Tx). In 2012 in Sweden [2], 2,894 patients had HD, 132 patients had HHD, 786 patients had PD, and 5,040 patients had kidney transplants. At chronic end stage renal disease (ESRD) it becomes clear how the disease and its treatment limit the patient's quality of life [3–5]. For example, the patient's life revolves around dialysis treatments that he/she receives several times and for many hours per week; the patient's food intake and social life are severely affected [4]. It is not only the patient's life that is adversely affected but that of the patient's relatives [3, 5, 6]. HD means that the patient goes to hospital, for example, three times a week for four hours each time, to get the blood purified with help of a dialysis machine. It is the availability of dialysis machines, staff, and the patient's energy and time that limits the number of dialysis treatments per week. The more dialysis patient receives the better it is for the patient's health.

HHD, that is, haemodialysis in the patient's own home, has become increasingly common in recent years [7]. It makes it possible for the patient to have daily or nightly dialysis in his/her own home. The patient's own resources are utilized and the patient can choose when to have their treatment. All patients who are sufficiently motivated can be trained to self-manage and carry out HHD. It usually takes 6–8 weeks at a clinic to train patients. In New Zealand approximately 25% of the total dialysis population has HHD [7]. In Sweden, the proportion of HHD-patients is approximately 3% of the total dialysis population [7]. A study in the USA highlighted the effects of HHD [8]; the results show increased patient well-being and better relationship with partners compared to when the patient started the HHD treatment. According to a study from Minnesota [9], frequent dialysis reduces the chance of cardiovascular disease and improves quality of life; however, it remains unclear whether it influences the survival chances of severely ill patients. In Canada, HHD-patients show physical and mental improvement [10] as they felt they had more control over their lives and their negative experiences of the illness decreased. This study found that the health professionals' support was important for HHD to be successful. Rygh et al. [11] have revealed that when patients are choosing their preferred form of treatment, the information they get from other patients about HHD influences them more than information they get from healthcare providers (a.a.).

Nurses who care for patients that are chronically ill and in need of dialysis treatment give a lot of support and information to educate the patients. Pagels et al. [12] have studied nurse-led patient education and found that the patients who are well-informed choose to start dialysis in PD and HHD to a greater extent than patients who have not received sufficient information on treatment options. The results also highlighted that the patients that chose HHD estimated their own care ability to be higher than before they started HHD (a.a.). Patient education and the patient's learning process significantly affect adaptation to the disease and play an important role in the treatment [13]. According to Swedish law, patients should be allowed to be as involved as they wish in their treatment [14]. However, patients and healthcare providers often have different perspectives on what the most suitable care is and what the patients need to learn [15]. Berglund [16, 17] explained that it is a big challenge to learn how to live with a chronic disease. Learning is described as a complex ongoing process that involves the whole person, in physical, mental, and life situations. It involves understanding the consequences of choices made in daily living and being able to see opportunities to influence one's life in terms of the illness and treatment (a.a.). HHD is a treatment alternative of increasing popularity and medical studies show that HHD positively affects the patient's health. Despite its growing popularity, there remain few studies that fully explore the advantages and disadvantages of HHD from patients' perspectives. Therefore, the aim of this study is to fill this gap.

## 2. Method

A qualitative approach was selected to suit the aim of the study. According to Henricson and Billhult [18], new knowledge of a phenomenon can be developed with the help of qualitative data such as interviews or written narratives. According to Dahlberg et al. [19], written narratives focus on an episode of the informant's experience, which in one way or another highlights the phenomenon in focus. The advantage of written narratives is that they are often focused on a given topic; that is, one can avoid the informant dwelling on material that is not relevant to the given experiences or perceptions, which is common in interviews. The weakness of written narratives is that the researcher has less opportunity to supplement the information than during an interview. When the informant describes a lived experience, the focus is usually on a critical situation. Asking for both a positive and a negative narrative allows for a more balanced picture (a.a.).

### 2.1. Participants and Data Collection

The sample consisted of all patients from a hospital in Sweden who have HHD (*n* = 11). Permission was requested from the hospital director before information letters were sent to the informants. Data collection was conducted from June 2013 to August 2013. The participants were informed first by letter and then orally. They were asked to write narratives about their positive and negative experiences of HHD. They were asked to describe one or two situations from their everyday life with HHD. They were told to describe the situations as accurately and fully as possible with the thoughts and feelings they had in the situations.

Nine of the eleven patients were included in the data collection and two chose not to participate. There were five men and six women, between 30 and 70 years old. The participants had different social situations: some lived alone, some with partners, and some had children at home. For some the relative was working and for others the relative was retired and thus at home more frequently. All lived in their own house.

### 2.2. Data Analysis

A qualitative analysis of the narratives has been used. The analysis can be described as a process, starting with the whole, analysing its parts, and followed by the rebuilding of the whole with the aim of reaching an understanding of the phenomenon [19, 20]. The analysis starts with the initial whole, where the researcher becomes familiar with the text. The familiar release phase involves the researcher reading the text as a whole and getting an early understanding of it. The narratives were read in their entirety several times until the researchers could easily identify each text. Dahlberg et al. [19] argue that when the researcher can easily describe the different narratives, it is time to move on. The analysis phase involves the researcher using a creative interpretation to get a deeper understanding [19]. The focus of the reading is directed to the parts, which means seeking units of meaning: a word, a sentence, or a longer piece of text. The meanings were sorted into groups. Based on this analysis of the texts, five themes were developed. Quotes from the narratives accompany the themes to make it possible for the reader to understand how the narratives have been construed.

### 2.3. Ethical Considerations

The ethical considerations for this study are based on ethical principles of research outlined in The Declaration of Helsinki [21] and Swedish law [22]. Based on these ethical principles, the requirements of information consent, confidentiality, and use have been applied. All participants received a letter with information about the study. The participants were guaranteed confidentiality and were informed that participation was voluntary and that at any time they could opt out without any consequences. The letter contained two envelopes, one for the written narratives and one for the consent. They were also informed that no information from their medical journals would be used. The narratives that were sent to us with signatures were made anonymous. Names of individuals, professionals, hospitals, and locations were changed so that the informants could not be identified.

### 2.4. Findings

The results describe patients' experiences of having HHD. The results are subdivided into five themes: freedom to be at home and have self-control of the treatment, feeling of being alone with the responsibility, changes in the home environment, needs of aids and safety, and well-being with HHD. Explanatory quotations are presented along with each main theme.

### 2.5. Freedom to Be at Home and Have Self-Control of the Treatment

The freedom that HHD brings is described as both a positive factor and negative factor. It is a freedom that gives the patient self-control of the treatment. The physician gives the patient an ordination of dialysis; it consists of a number of treatment hours that the patient needs in a week. With a dialysis machine at home the patient can do the treatment whenever he/she wants, without needing to adapt to the clinic's opening times or making appointments for treatment. This was a positive experience for the patients. Dialysis in a hospital means being forced to adapt one's life in keeping with timetabled treatment. HD at home means having the freedom to decide when is suitable to do the treatment and how often or how long each treatment should be. Consequently, patients have more freedom in planning their everyday activities on the basis of what they want to do and they can fit the treatment around these activities. One patient describes this freedom in the following way:

*“You do not need to adapt your day to the clinic's opening time and access of place. You can alone choose which day in the week you have dialysis. Further, you can decide from day to day when in the day or night you will start the dialysis.”*

HHD gives patients the freedom to have the treatment at night. Those who have night-time dialysis find that they can spend their days more freely than if they have the dialysis during the day. Night-time dialysis also makes them feel less tired.

The freedom also means being able to control the treatment in keeping with changes in the weather and the seasons. Patients find it difficult to do dialysis indoors when the sun is shining and they would rather be outside. In the summer, many choose to do the dialysis in the morning. In that way they can get out more of the day. The freedom in that case is to decide when the treatment least interferes with activities. This feeling of freedom and self-control is described by one of the patients in the following way:

*“I don't yell HURRAY each time I do dialysis, but I yell hurray because I will and can do it at home.”*

The freedom is also seen in the ability to rest directly after the dialysis treatment in the own home. HHD means that the patients do not have to travel to the hospital for treatment. They describe their earlier experiences of journeys to and from the hospital as time-consuming, tedious, and boring. It is also bothersome to be forced to pursue conversation with the taxi driver when they are tired and want to get straight home. HHD thus offers more control over their time and more comfort after treatment.

HHD also means that the patients minimize the risk of infection as they do not have to share a room with other patients. A patient describes this as his/her reason for choosing HHD as follows:

*“When I decided to do home haemodialysis, it was the freedom that decided me. To lie for 4-5 hours in a big room together with many others is not my ideal.”*

The freedom to be at home and manage the treatment alone is, however, limited. Sometimes technical problems arise with dialysis equipment. Engineers cannot always repair the machine at once and then patients have to go to the hospital for treatment, which they find stressful. Having to go to the hospital when problems with the AV-fistula arise is viewed as negative, particularly if the period becomes too long. The patients would like a better backup service so that they do not have to go to the hospital in the case of technical problems. Some patients find it unnecessary to do dialysis in hospitals in connection with the dialysis monitoring and the physician's appointment. They describe it as a routine from the dialysis unit. They would rather only see the physician and then go home.

The freedom to manage the treatment independently is linked to the home, but life also includes going on holidays. Patients find travel difficult because the guest clinic offers limited times for dialysis that are dependent on the clinic's facilities. This hinders some of the patients from travelling. A patient describes this feeling as follows:

*“Sometimes, it feels heavy why this disease will affect precisely me. When friends go on vacation or do something else pleasant, I have no possibilities to follow. That makes me sad and everything feels very troublesome.”*

A portable haemodialysis machine makes it possible for the patients to travel. This machine gives the patient the freedom to do the dialysis wherever they stay (hotel or apartment), and the patient's knowledge can strengthen his/her feeling of freedom. A patient describes it as follows:

*“Obviously there is a lot of planning before travel with a dialysis machine. Well on-the-place, I can enjoy my travel and still be free to do the dialysis when I want to. I do not need to adapt myself to a hospital on the vacation.”*

The freedom to be at home and have self-control of the treatment is central to the patients' experiences of HHD. They can make many choices and thereby control their life situation with the treatment, but the freedom is limited by the treatment itself and technical or medical problems.

### 2.6. Feeling of Being Alone with the Responsibility

Experiences of having HHD also involve a feeling of being alone with the responsibility for the treatment. The patients describe that they often are alone at home when the treatment is on-going. Their partner does other things when the patient has their treatment. This can sometimes be seen as more negative than having dialysis in the hospital where health professionals or other dialysis patients talk with them.

Furthermore, because they are alone during the treatment, the patients worry about complications. Complications can be caused by the patient or by a technical error on the dialysis material or machine. In stressful situations, for example, because of lack of time, it is easier to make a mistake or forget to do something and this error could endanger the patient's life. A patient describes how he/she has learned to handle stress in the following way:

*“I learned to NEVER go from my procedures. The time I would have saved by doing different things was only seconds, if even that! After this event, there is always a nervousness to do the same thing.”*

The feeling of being alone with the responsibility becomes apparent in the descriptions of complications. For example, blood loss can occur and go undetected. The patients describe how they must sort out situations like this by themselves. They also describe their fear of getting hypotension and losing consciousness and therefore not being able to call for help. This creates anxiety and sometimes the patient's blood value or blood pressure needs to be checked in hospital. Some patients feel cautious and insecure when the dialysis clinic is closed. A patient describes the feeling of loneliness when he/she is unable to reach the dialysis nurse or technician in the following way:

*“We are not physicians or health professionals and if something happens late in the evening it feels extremely lonely; where do you turn?”*

The feeling of being alone with the responsibility increases when the nurses give various directives concerning hygiene requirements, for example, how the patient will deal with AV-fistula/CDK at the start and close of the treatment. The patients become afraid to make mistakes and feel alone with the responsibility.

### 2.7. Changes in the Home Environment

The physical environment at home changes when the dialysis machine is installed. Patients explain that the dialysis machine, water cleaner, dialysis materials, and empty wrappings require a lot of space at the home. Their otherwise peaceful home environment is disturbed by the sound that the equipment causes. Most of the participants view having the equipment at home positively, despite realizing that the environment is changed. One patient describes how he has placed his dialysis equipment to keep it close at hand but in a way that it does not intrude too much on the home space:

*“I have arranged a wardrobe in connection with the bedroom where the machine, water cleaner and dialysis material is near to hand.”*

The environment includes the home situation in many ways and one of which is the family finance. Patients describe how increased cost of electricity and water involved in running the dialysis treatment affected the family finances.

Furthermore, as the social environment changes, the participants describe how they have more time with their families and more time to socialize. They can plan their time and attend family activities; this is a positive experience that stems from HHD.

### 2.8. Need of Support and Security

Patients who have HHD need support and security. Above all, the patients call attention to how important it is to have support from relatives and health professionals at the dialysis clinic. Some patients who have a relative at home experience great support:

*“I have incredible support from my relative when the machine sounds and malfunctions. I might be worried but he is a lot calmer, so I also calm down.”*

The support brings a feeling of safety especially when complications arise during the dialysis treatment at home. The patients also feel secure when they get advice and support from nurses and technicians by phone when something fails. The patients experience lack of support from the primary health care units when they want to leave blood samples at the nearest health care unit and the health professionals do not know how to take care of them. On the other hand, they can refer to staff on the dialysis ward that can help the health professionals at the primary health care. A patient describes this experience in the following way:

*“If something fails, there is always some health professionals from the dialysis ward who can help and advise me on the telephone. Nothing is impossible.”*

The patients experience support and a feeling of safety when they visit the dialysis ward. Here they can get information about their blood values and get help with the adjustment of medicines.

During treatment on a dialysis ward, a number of different nurses make the perforation of the blood access. Unfortunately that can cause problems with the blood access, which is so important for the patient's life. This is an important lifeline; without it the patient cannot get the life support treatment. With HHD the patients do the perforation of the access themselves, and they see this as a clear advantage of HHD. It creates a feeling of safety to have the control over who perforates and that it is done in the same way every time.

### 2.9. Well-Being through HHD

Having HHD means that the patients feel better and stronger compared to when they had their treatment in hospital. Through HHD the patients have the possibility to get dialysis more continually, that is, for six to seven times a week and for several hours than they might have had in hospital where three to four times a week is a common treatment regime. The more continually dialysis that HHD brings they believe is the reason that they find out that they feel better. Some patients describe how they “have got their life back.” They have the energy to do things they have not done for a long time. The identification with a chronic disease has decreased with HHD. A patient describes this in the following way:

*“The dialysis machine always stands at home and reminds about the disease. For me it is very important to not let the disease take over. It is a part of me but I don't identify myself with it, what I unfortunately often have seen happen to others who have a disease.”*

The patients describe how they feel better and have fewer restrictions when it comes to fluid and food intake. They can allow themselves to eat what they desire and this is something that increases a general sense of well-being. The patients that have nocturnal HHD experience less strain on their bodies; they feel less tired. This is described by a patient in the following way:

*“After hesitation I went over to nocturnal dialysis. This dialysis method functioned very well because the treatment becomes less straining for the body and I could spend my days in a more free way. I had my time at my disposal and I was not tired.”*

Some patients remember the positive feeling of doing the first dialysis treatment at home, with a nurse as support. They also describe the courage they had to cope with the treatment and how good it felt. Their self-confidence increases over time and they dare to do treatment even when they are alone at home. The responsibility is big, but they grow with the assignment. Although incidents during dialysis can happen, the patient has the courage to be able to cope with the situation. A patient described it as follows:

*“You control your own life and take responsibilities for your health. It is a big, heavy and weighing responsibility.”*

The courage also gives the patients fortitude to stand firm when they encounter obstacles, for example, when they are in contact with other health care providers. In this situation, the patient can feel proud of his/her knowledge and skills. One patient said, “They have never heard of someone being able to do it like this”.

## 3. Discussion

The results in this study show that patients experienced a sense of freedom from being at home and controlling the treatment, that is, having the freedom to choose when and how the treatment would be done. Todres et al. [23] and Dahlberg et al. [24] have stressed the value of life-world-led care. This provides a direction for care and practice that is intrinsically and positively health focused and where the patients are seen in their contexts as a whole with physical, psychological, and social dimensions. Life-world-led cares support the patient to balance between the vulnerability and freedom that the illness and treatment gives. The results show the importance of the freedom to plan rest and treatment and find a healthy life rhythm that HHD gives. Polaschek [25] notes that HD in hospital requires the presence of the patients in keeping with the hospital's schedule. The travel and treatment can take an entire day. HHD is beneficial because the patient chooses when he/she wants to do the treatment (a.a.); this is in line with the results of this study.

Some patients describe a sense of being in captivity despite availing of HHD. The physician prescribes a certain number of hours of dialysis per week. They find it difficult to motivate themselves when the weather is good or they have plans to do something nice. The patients feel lonely and isolated because of their chronic disease and the need for HHD. The freedom to be at home and have self-control of the treatment can thereby be understood as both a positive and negative factor. This can be related to Dahlberg's and Segesten's [26] theory about living with ill health; they describe the determination to live with a prolonged or incurable disease, something that you cannot release yourself from and that greatly limits the daily life (a.a.). Berglund [17] argues that it is important to understand this; that is, it is important to realize what is possible and impossible to affect in terms of the treatment or their way of life.

The results that show patients' feelings of being alone with the responsibility are related to concerns about acute complications that can happen during treatment. Patients feel anxious and isolated despite having the treatment at home for a long time. Wong et al. [27] describe how patients have learnt to carry out nocturnal HHD, despite worrying about unexpected incidences and medical responsibility. The results in our study show an increased self-confidence over time and patients feel confident enough to do the treatment even when they are alone at home. This indicates that the feeling of being alone with the responsibility allows the person to grow with the role.

The results show that the patients need support and stability. The patients feel insecure about acute dialysis complications when they cannot reach health professionals by telephone. This is in line with Xi et al.'s [10] results that found that the health professionals' support is important for HHD patients. They highlight the importance of being able to call the hospital and get help with problems with the treatment. Polaschek [25] suggests that the nurse plays a key role in helping people live with chronic conditions. According to Öhman et al. [28] and Berglund [3], living with uncertainty is central to people that live with chronic diseases. They are forced to live a life they have not chosen and this often results in feelings of uncertainty, loneliness, disappointment, and fear. According to Rygh et al. [11], telemedicine/web camera can support the patients if complications arise with HHD. By web camera, the patient can be offered support and instructions; this may be something to develop in the HHD treatment.

The result shows that the patients' feelings of well-being increased with HHD. They have more energy to do things that they have not been able to do for a long time. The patients also describe how they are getting greater courage to stand up for themselves at setbacks. They also have an increased sense of responsibility and have developed knowledge and understanding of their disease and treatment. Dahlberg and Segesten [26] describe health as the ability not only to do things that are needed in life and of importance for you but also to make choices and take responsibilities. Health is to have balance in life and to feel vitality (a.a.). Wise et al. [8] found that HHD-patients tend to feel better and have an increased sense of well-being. Xi et al. [10] recorded physical and mental improvements in HHD patients. Fatigue decreased and the patients could begin to work again (a.a.). Berglund [3] argues that genuine learning on an existential level can contribute to increased health and well-being, and knowing how to handle HHD can be seen as learning on this level. HHD gives the patient the possibility to make conscious decisions and take charge of his/her life on the basis of those possibilities he/she has. We understand this as increased well-being.

## 4. Method Discussion

A qualitative analysis of the material was relevant for the study because the patients' lived experience of HHD was examined. Patients with experience of HHD wrote narratives about the phenomenon. The advantage of written narratives is that the informants themselves have chosen which information they provide to us. In this study, the participants knew the researchers so the choice of data collection method was also an ethical issue. According to Dahlberg et al. [19], a disadvantage with written narratives can be that the researcher has less possibility to complement the information than he/she does with, for example, an interview. While we agree with this argument, after considering the benefits and drawbacks of each method of data collection, we concluded the written narrative was the most suitable under the circumstances. As the informants know and depend on us as treating nurses and we have preunderstanding of the situation, we had to choose an appropriate method of data collection. One common preunderstanding that was documented before the start of the study was reconsidered after the study; the results have revealed many aspects of the treatment that we were previously unaware of. Furthermore, the preunderstanding has been reflected on and discussed with our supervisor and colleagues at the clinic, as suggested by Henricson and Billhult [18].

## 5. Conclusion

The results show not only that the patients feel HHD gives them the freedom to be at home and have self-control of the treatment but also that they feel self-reliance on the treatment. The patients feel physically and mentally better because they can dialyse themselves more often. HHD requires courage to overcome the fear of making mistakes. The knowledge and experience that patients with HDD have give them more confidence and opportunity to discuss the treatment and make demands on their care. The research also found that it was beneficial to the patients' well-being to not have to travel to a hospital for dialysis. However, the research found some negative aspects of HHD; the patients sometimes lack support and a sense of stability from dialysis personnel.

Patients that suffer from chronic end stage renal disease have to be well-informed and supported when they choose treatment as PD, HD, or HHD. An important part of this information should be based on patients' experiences of living with the treatment. The findings in this study can increase understanding of what HHD is from patients' perspective and are useful in the information of treatment option.
